# Supplementary material for: Staphylococcus haemolyticus and Providencia vermicola Infections Occurring in Farmed Tilapia: Two Potentially Emerging Pathogens
Source: Animals (Basel). 2023 Nov 30;13(23):3715. doi: 10.3390/ani13233715 (PMC10705304; doi:10.3390/ani13233715)
Supplement: Supplementary file 1 [file animals-13-03715-s001.zip › animals-2693810-supplementary.pdf]

Supplementary material

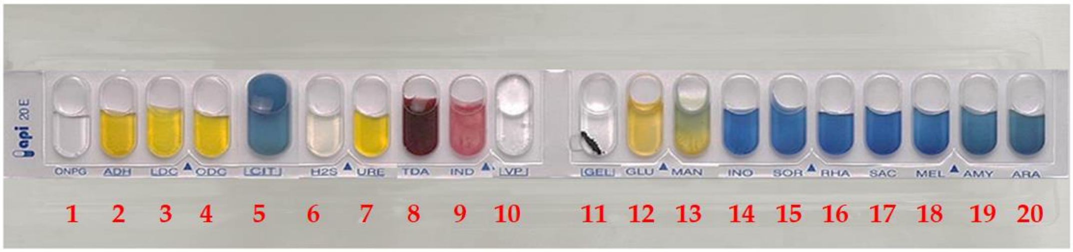

Supplementary Material S1. Representative image of the results of biochemical tests of the five Gram (-) isolates through the miniaturized API® 20E system. According to the manufacturer's references (bioMérieux), the isolates were considered to belong to the *Providencia* genus. 1: ONPG [-], 2: ADH [-], 3: LCD [-], 4: ODC [-], 5: citrate [+], 6: SH2 [-], 7: urea [-], 8: TDA [+], 9: indole [+], 10. Voges-Proskauer [-], 11: gelatin [-], 12: glucose [+], 13: manitol [+], 14: inositol [-], 15: sorbitol [-], 16: rhamnose [-], 17: saccharose [-], 18: melibiose [-], 19: amygdaline [-], 20: arabinose [-]

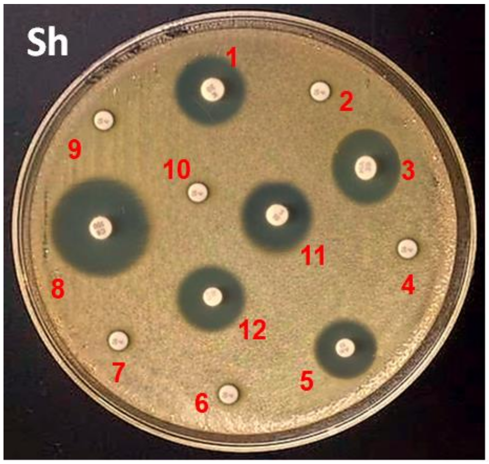

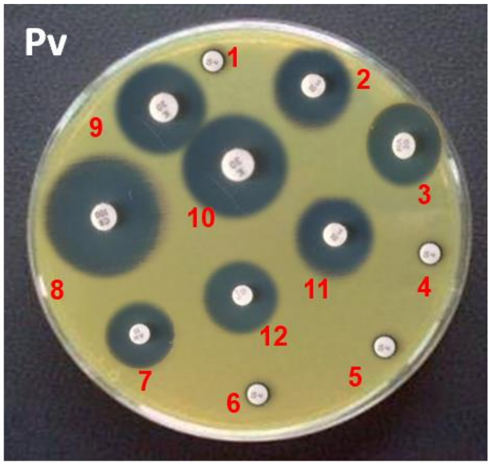

Supplementary Material S2. Qualitative antimicrobial susceptibility testing of bacterial isolates using the disk diffusion method in Mueller-Hinton agar. SH: *S. haemolyticus*; Pv: *P. vermicola*. 1: chloramphenicol, 2: ofloxacin, 3: cotrimoxazole, 4: oxytetracycline, 5: penicillin G, 6: erythromycin, 7: ciprofloxacin, 8: enrofloxacin, 9: oxolinic acid, 10: nalidixic acid, 11: florfenicol, 12: fosfomycin. In cases where a gray halo was observed around the disc as an indication of inhibition of bacterial growth, it was considered antimicrobial sensitivity. In cases where the inhibitory halo was not formed, it was interpreted as antimicrobial resistance.
